# Is lack of habituation a biomarker of migraine? A critical perspective

**DOI:** 10.1186/1129-2377-16-S1-A13

**Published:** 2015-09-28

**Authors:** Filippo Brighina, Giuseppe Cosentino, Brigida Fierro

**Affiliations:** Dipartimento di Biomedicina Sperimentale e Neuroscienze Cliniche (BioNeC), Università di Palermo, Palermo, Italy

Processing of sensory stimuli has been supposed to be dysfunctioning in migraine. A basis for such abnormality has been identified in a defective ability to habituate to repetitive sensorial stimulation. Habituation, i.e. the way the nervous system attenuates response to repeated non noxious stimuli is a fundamental function of sensory systems, that allows appropriate adaptation of neural responses to the relevance of incoming stimuli. In humans, habituation can be studied by evoked potentials where it is indexed by a reduction of amplitude of the evoked response to repeated stimulation. After the first evidence by Schoenen et al in 1995[1] of reduced habituation to visual evoked potentials in migraine the defect was confirmed in other studies, not only with visual stimuli, but also with other sensory modalities (acoustic, somatosensory) and even with nociceptive stimulation. For such a consistency lack of habituation has been considered a neurophysiological hallmark of the disease. However, critical aspects concerning this statement have been recently raised because the requirement for a disease hallmark appeared not to be met by lack of habituation[2, 3]. A disease hallmark should be intrinsic to the pathophysiology of a disease and as such ubiquitous or quite so and specific for that disease. This however, does not seem to be the case for lack of habituation in migraine. Some authors indeed were not able to find defective habituation in this disease and recently relevant criticism about this dysfunction has been raised by the group of Sand and Omland[2]. These authors indeed applying a different methodological approach (with a blind procedure for both VEP recording and analysis) in a series of studies (exploring a wide range of stimulation parameters) were not able to replicate, at least for visual modality, the habituation defect and attributed this to a likely expectancy bias not adequately controlled in previous studies. Moreover, dysfunctioning habituation is not specific for migraine as it has been found in several other diseases ranging from chronic pain states, to deafferentation diseases like tinnitus, or degenerative pathologies like Parkinson's disease. Thus, more than strictly related to migraine pathophysiology, lack of habituation could represent a more general marker of neural dysfunction that migraine can share with several other diseases.
